# Simulation-based randomized trial of medical emergency cognitive aids

**DOI:** 10.1186/s13049-022-01028-y

**Published:** 2022-07-11

**Authors:** Timur Sellmann, Samer Alchab, Dietmar Wetzchewald, Joerg Meyer, Tienush Rassaf, Serge C. Thal, Christian Burisch, Stephan Marsch, Frank Breuckmann

**Affiliations:** 1 Department of Anaesthesiology and Intensive Care Medicine, Bethesda Hospital, Heerstrasse 219, 47053 Duisburg, Germany; 2 Department of Anaesthesiology I, Witten-Herdecke University, Witten, Germany; 3 Department of Internal Medicine 1, Herz-Jesu-Hospital Dernbach, Dernbach, Germany; 4 Institution for Emergency Medicine, Arnsberg, Germany; 5 Department of Anesthesiology, Operative Intensive Care Medicine and Pain Therapy, St. Marien-Hospital, Muelheim an der Ruhr, Germany; 6 Department of Cardiology and Vascular Medicine, West German Heart and Vascular Center Essen, University Duisburg-Essen, Essen, Germany; 7 State of North Rhine-Westphalia / Regional Government, Duesseldorf, Germany; 8 Department of Medical Intensive Care, University Hospital of Basel, Basel, Switzerland

**Keywords:** Cognitive aid, Medical education, CPR, Simulation, Patient safety, Patient care

## Abstract

**Background:**

Medical emergencies are complex and stressful, especially for the young and inexperienced. Cognitive aids (CA) have been shown to facilitate management of simulated medical emergencies by experienced teams. In this randomized trial we evaluated guideline adherence and treatment efficacy in simulated medical emergencies managed by residents with and without CA.

**Methods:**

Physicians attending educational courses executed simulated medical emergencies. Teams were randomly assigned to manage emergencies with or without CA. Primary outcome was risk reduction of essential working steps. Secondary outcomes included prior experience in emergency medicine and CA, perceptions of usefulness, clinical relevance, acceptability, and accuracy in CA selection. Participants were grouped as “medical” (internal medicine and neurology) and “perioperative” (anesthesia and surgery) regarding their specialty. The study was designed as a prospective randomized single-blind study that was approved by the ethical committee of the University Duisburg-Essen (19-8966-BO). Trial registration: DRKS, DRKS00024781. Registered 16 March 2021—Retrospectively registered, http://www.drks.de/DRKS00024781.

**Results:**

Eighty teams participated in 240 simulated medical emergencies. Cognitive aid usage led to 9% absolute and 15% relative risk reduction. Per protocol analysis showed 17% absolute and 28% relative risk reduction. Wrong CA were used in 4%. Cognitive aids were judged as helpful by 94% of the participants. Teams performed significantly better when emergency CA were available (p < 0.05 for successful completion of critical work steps). Stress reduction using CA was more likely in “medical” than in “perioperative” subspecialties (3.7 ± 1.2 vs. 2.9 ± 1.2, p < 0.05).

**Conclusions:**

In a high-fidelity simulation study, CA usage was associated with significant reduction of incorrect working steps in medical emergencies management and was characterized by high acceptance. These findings suggest that CA for medical emergencies may have the potential to improve emergency care.

**Supplementary Information:**

The online version contains supplementary material available at 10.1186/s13049-022-01028-y.

## Background

Medical emergencies are stressful and complex events that require rapid and coordinated care in a time-critical setting. Guideline-adherent management has been recognized as an important outcome-relevant factor (1;2). Data concerning unexperienced physician’s management of medical emergencies showed potential deficits in the work-up (3) and thus, unexperienced teams often fail to properly manage medical emergencies. Room for improvement was identified during emergencies (e.g. cardiac life support) as well as in the early phase after a critical event (2;4). Checklists and protocols have long been accepted in high-risk industries (e.g., aviation and nuclear power) as a tool to aid performance during rare and unpredictable critical events and as components of the safety culture translated to medical practice (3;5). The use of surgery-specific safety checklists during routine operative care have been associated with significant reductions in morbidity and mortality (3) and has thus become a standard of care (6;7). In medicine, the use of checklists, amongst others, has been associated with improved patient outcomes in circumstances where the checklist can be used for largely linear processes such as the preparation for central venous catheter insertion (5). While the usage of checklists is thought to improve care, they simultaneously bear the risk that clinicians may feel restricted (8), and, if wrong checklist are selected they may influence overall team-performance as well. In addition to checklists, there are a number of other tools and instruments designed to work with in medicine. As the text-based algorithms used in this study do not contain any obvious actions or criteria to "check off", we chose the term “medical emergency cognitive aid” (MECA) instead in order to ensure transparency with readers.

In the current study, we sought to analyze the effect of unknown MECA for multi-professional teams of physicians in a simulated setting, using a high-fidelity medical simulation to facilitate a structured observation of the medical emergencies. We tested the hypothesis that MECA would significantly reduce the number of incorrect process steps in the work-up of medical emergencies, expressed as absolute and relative risk reduction in an intention to treat and per protocol analysis of a randomized, controlled trial involving physicians (trainees) from different professions (e.g., anesthetists, surgeons, neurologists, internists) as well as different levels of experience.

## Methods

### Study design

The current analysis was performed during five educational critical care medicine courses from end of 2019 to mid of 2020. The study was designed as a prospective randomized single-blind study that was approved by the ethical committee of the University Duisburg-Essen (19–8966-BO) and published in a national clinical trial data register (DRKS00024781; www.drks.de).

## Participants and setting

**Fig. 1:**
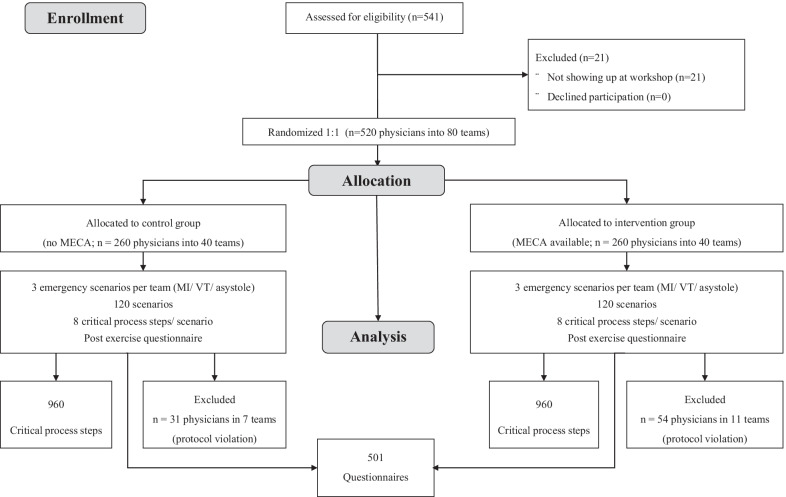
CONSORT Flow chart showing the test set-up. MECA = medical emergency cognitive aid. Due to the experimental procedure, neither an exact assignment of questionnaires/ participants (MECA used?) nor an exact assignment of participants/ MECA (when used?) is possible

On a regular basis, the “Arbeitsgemeinschaft Intensivmedizin”, Arnsberg, Germany (http://www.aim-arnsberg.de) organizes educational courses for physicians from all over Germany working in intensive care medicine. For most participants (usually residents in their 2nd to 3rd year of clinical practice in internal medicine, anesthesia, surgery, or neurology) this course represents their first post-graduate training course related to intensive care. Interdisciplinary plenary lectures about medical emergencies of all kinds were held prior to the start of the workshops as part of the course content. Course participants were offered to take part in voluntary simulator-based emergency training. Teams of three to six physicians each were equally randomized (single blinding) with research randomizer (www.randomizer.org) to perform simulated emergency scenarios with or without a dedicated MECA folder. Structure and contents of the MECA were briefed immediately prior to the emergency training. We used checklists adapted by JM and TS in their institution from a work by Arriaga et al.in 2013 (3). The MECA folder contained a total of 10 life-threatening medical emergencies, including cardiac arrest, bradycardia, difficult airway, hypoxia, bleeding, pulmonary embolism, anaphylaxis, in German language, with three determined to select by the course format (see Additional file 1: Appendix A). The degree of implementation of critical process steps was determined (CONSORT flow chart; Fig. 1). Written informed consent was obtained for study participation and video recording of the emergency scenarios. Additionally, the participants were asked to fill in a questionnaire on the topic.

## Scenario and checklists

All participants received a standardized introduction to the simulation environment and were made familiar with the manikins used and the medical equipment available. For only those groups allocated to use checklists, the folder including all MECA was handed over directly before starting emergency simulation. Teams consisted of three to six physicians each. Team members were then informed that their role during the following scenarios was that of an in-hospital rapid response team (RRT) called to the hospital’s emergency department (ED). The core RRT consisted of at least three doctors (anesthesiologist, internist, and surgeon, all random and not necessarily corresponding to the real profession) and a nurse (played by another doctor). The allocation of roles between MECA lecturer leader and team members was open free and up to the teams themselves.

The patient introduced by an ED physician was either suffering from a myocardial infarction deteriorating into ventricular fibrillation, suffering from hemodynamic unstable ventricular tachycardia (VT) or bradycardia deteriorating into asystole caused by hyperkalemia. All rhythm disorders could be diagnosed on the display of the defibrillator simulator once attached. The study period ended with return of spontaneous circulation after the third defibrillation in the first setting, after termination of ventricular tachycardia by cardioversion in the second setting and after treatment of hyperkalemia in the third setting. Manikins were operated by trained tutors who were instructed to refrain from any intervention until the end of the study period. Except for this study-specific instruction, scenarios with or without MECA were identical. “Critical action items” (labelled red and asterisked for a better traceability in Additional file 1: Appendix A) were predetermined by interdisciplinary consensus on the basis of binary assessability (performed/ not performed). In case of lengthy scenarios it was up to the tutor to schedule them on time if necessary.

## Structured survey assessment

Following the scenario, participants were asked to complete a structured questionnaire. Since checklists or cognitive aids are already more or less widely used in medicine in Germany, all participants (i.e., also those randomized to the non-MECA group) were asked about any previous experience with checklists. In addition to basic demographic data (age, gender, medical specialty) and prior experience (general, intensive care and emergency medicine work experience), prior use of checklists or related tools as well as statements about the usefulness of MECA were evaluated. The latter parameters were stratified by using a 5-scale Likert scale. Participants were also asked to indicate their position within the scenarios (team member or leader, checklist lecturer). To allow better comparability, specialties were added up into "medical" (internists and neurologists) and “perioperative” (surgeons and anesthesiologists), a division that has been done comparably before [9, 10].

### Video analysis of checklist usage

Interrater reliability was assessed in the beginning of the analysis and randomly by two independent reviewers (SA, FB). In case of disagreement or uncertainty among reviewers regarding adherence by the team to a key process, these were resolved by bringing in external additional experts (assessment by a senior anesthesiologist, or an emergency physician; FB, TS, SA) and concluded by a joint consensus. Due to structural and technical requirements, video analysists could not be blinded. The use of MECA by a participating team was not blinded either. Start for the timing of all events was defined to be the start of handover to the team. The correct choice of MECA within the intervention arm was evaluated binarily (yes or no). Each scenario was basically evaluated with eight critical work steps. These were derived from evidence-based guidelines and also scored in a binary fashion. Usage of wrong MECA resulted in zero points. In case of premature termination of a scenario (e.g., prior third defibrillation before application of amiodarone or after first cardioversion before increase of cardioversion energy), the total number of critical steps to be achieved was reduced accordingly. Teams committing relevant protocol violation (i. e. mandatory interference by the tutor) were excluded.

In an additional per protocol analysis, the following scenarios were excluded from evaluation: teams choosing the false MECA, teams using no MECA even though provided, and teams committing any protocol violation (e.g., interference with the tutor).

### Endpoints

The primary outcome measure was the error rate reduction of critical work processes for each emergency. In the overall evaluation, all critical steps (including misapplication of MECA) were assessed and thus the relative and absolute risk reduction was calculated. Risk reduction of each individual scenario (myocardial infarction, hemodynamically unstable VT, hyperkalemia-induced asystole) was determined separately. All analyses were performed for the 240 scenarios and for the additional per protocol analysis.

Secondary endpoints included basic demographic data, a survey regarding participants` perceptions of the usefulness and clinical relevance of the MECA, prior medical and emergency care knowledge, pre-existing experience with MECA, influence of experience and specialization, quality of the MECA and the emergency scenarios and overall acceptance as well as accuracy of checklists usage. Additionally, encouragement of the participants` subjective sense of security and the influence of the MECA on the interaction during the scenario were evaluated.

### Statistics

All data were analyzed on an intention to treat as well as per protocol basis. Following testing for standard deviation (SD), data were expressed as means ± SD unless otherwise stated. Student’s t-test and Chi square test were applied as appropriate. All reported p-values are two-sided, and a *p* < 0.05 was considered significant. Statistical analysis was performed using SPSS (version 22). Due to the purely observational character of the study, no power analysis was performed to determine a sample size.

## Results

### Basic demographic data

Overall 520 physicians took part in our study (52% female, 48% male) and 95% were less than 40 years of age. Previous experience was available in 48% for ICU work and in 17% for MECA use.

The participants, all from specialties related to emergency medicine, had a wide range of years of experience in their specialty (Table 1), with about a half (52%) being in the 2nd or 3rd year of their specialist training (residency). The relation “medical” (335 internists, 28 neurologists) to “perioperative” (anesthetists 85, surgeons 46) was 363 to 131. Seven participants belonged to “other” specialties (not explicitly specified). During the study about a third (34%) of the participants acted as team leader, 61% as team members and 5% as MECA presenters.

**Table 1:** Previous overall work and emergency care medicine knowledge of the participants

**Participants**			**Overall**	**Perioperative**		**Medical**		**Others**	p-value**
n*=501				**Anesthesia**	**Surgery**	**Internal Medicine**	**Neurology**		
				**n (%)**	**n (%)**	**n (%)**	**n (%)**	**n (%)**	
Experience	Total Years	≤ 1	69	15 (18%)	8 (17%)	43 (13%)	2 (7%)	1 (14%)	0.31
		2-4	373	61 (72%)	32 (70%)	253 (75%)	23 (82%)	4 (57%)	
		≥ 5	56	9 (10%)	6 (13%)	36 (11%)	3 (11%)	2 (29%)	
	Intensive care	Available	236	47 (55%)	15 (33%)	160 (49%)	10 (36%)	4 (57%)	1
	Previous medical emergency	EP	39	19 (19%)	3 (6%)	15 (4%)	1 (3%)	1 (9%)	**0.000004**
		PHTLS	12	6 (6%)	1 (2%)	3 (1%)	1 (3%)	1 (9%)	**0.006**
		Paramedic	26	8 (8%)	2 (4%)	12 (3%)	1 (3%)	3 (27%)	0.052
		AMLS	40	8 (8%)	3 (6%)	26 (8%)	3 (10%)	0 (0%)	1
		ALS	161	27 (27%)	13 (27%)	109 (31%)	8 (27%)	4 (37%)	0.66
	ER	Available	245	51 (60%)	22 (48%)	158 (47%)	10 (36%)	4 (57%)	0.06

Bold values indicate a* p*-value < 0.05 was considered as statistically significant

*ER *emergency room, *EP *emergency physician, *PHTLS *prehospital trauma life support, *AMLS *advanced medical life support, *ALS *advanced life support

^*^Total number of responding participants, number of answers may vary

**Chi square test was performed for “medical” and “perioperative”

A total of 80 rapid response teams participated in three directly consecutive medical emergency simulation scenarios each, adding up to 240 simulated events. Forty teams executing 120 scenarios received MECA (intervention group), whereas another forty teams served as control group without MECA (control group). In all scenarios, a maximum of 1920 critical process steps for analysis were available.

### Analysis of checklist usage

**Fig. 2:**
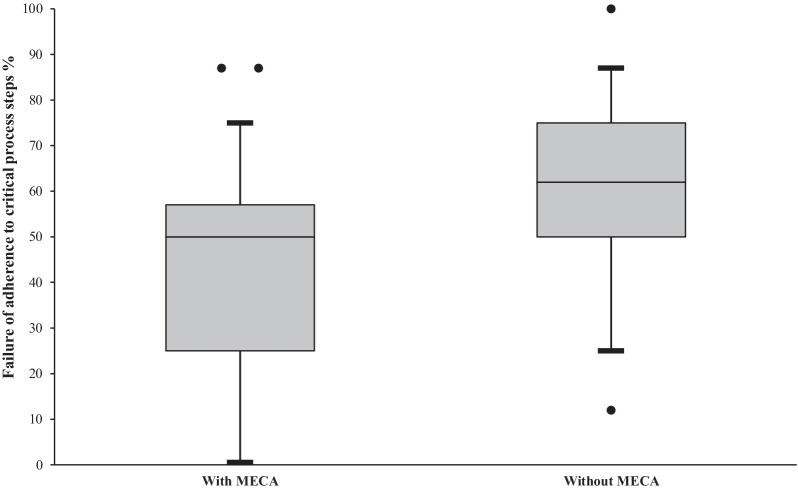
Completion of critical process steps – the use of MECA (medical emergency cognitive aids) resulted in 9% absolute and 15% relative risk reduction in the intention to treat and 17% absolute and 28% relative risk reduction in failure to adhere to critical work steps by per protocol analysis respectively (*p* < 0.05). Data are shown as median of failure rate, delineate the 5th and 95th percentile respectively. Dots displayed horizontally indicate values outside the 5th and 95th percentile. Left bar = intervention group (MECA available), right bar = control group (no MECA available)

In those scenarios with MECA, usage resulted in 9% absolute and 15% relative risk reduction of failure to adhere to guideline-adherent critical process steps. All teams had a lower failure rate for adherence to key processes when MECA were available. In those groups using MECA, failure of adherence was observed in 43% of critical process steps, in those groups without MECA critical process step were wrong or missing in 60% (*p* < 0.05, Fig. 2). The significant effect of MECA usage was seen for scenario one (CPR after MI) and three (hyperkalemia induced asystole) when stratifying the results according to the different scenarios.

### Per protocol analysis of checklist usage

Medical emergency cognitive aids were used in 63 scenarios. Forty-one scenarios were performed without MECA usage even though provided. Eleven scenarios were excluded due to protocol violation. In five scenarios, teams chose the wrong MECA. Using an incorrect MECA resulted in worse performance than groups that did not use a MECA at all, mainly based on a premature misinterpretation of the underlying cardiac rhythm. In the control group, seven scenarios had to be excluded due to protocol violation. All exclusions in both groups were due to the need for active interference by the tutor.

Per protocol checklist usage resulted in a 17% absolute and 28% relative risk reduction of failure to adhere to guideline-adherent critical process steps. The significant effect of MECA usage was also seen when stratifying the results according to the different scenarios (Table 2). Here, we saw a significant reduction of the failure rate per scenario for each checklist (*p* < 0.05).

**Table 2:** Effect of MECA use according to the scenarios (per protocol analysis)

Scenario type	Failure rate†		*p*-value‡
	With MECA	Without MECA	
	n (%)		
CPR after MI scenario	113/210 (54%)	221/332 (67%)	**0.004**
Hemodynamically unstable VT	25/117 (21%)	109/239 (46%)	**0.0002**
Hyperkalemia-induced asystole	74/168 (44%)	191/302 (63%)	**0.00006**

Bold values indicate a* p*-value < 0.05 was considered as statistically significant

*MECA *medical emergency cognitive aid, *CPR *cardiopulmonary resuscitation, *MI *myocardial infarction, *VT* ventricular tachycardia

^**†**^Failure rate was calculated as the number of critical steps that were not adhered to in the management of the scenario

^‡^P values were calculated in a model for clustering by team, scenarios in which the participants used the wrong MECA (e.g. bradycardia instead of asystole) were excluded (Scenario types were as follows: CPR after MI included ventricular fibrillation preceded by myocardial infarction, return-of-spontaneous circulation directly before fourth defibrillation; unstable VT included diagnoses, sedation and cardioversion, and hyperkalemia-induced asystole included bradycardia degenerating into asystole, discussing of potentially reversible causes and treatment of life-threatening hyperkalemia.)

### Analysis of structured survey assessment responses

A total of 501 participants (96%) completed the questionnaire. All participants rated the overall quality of the session as above average or excellent (score of 4 or 5, respectively). A total of 94% of the participants found the use of MECA reasonable. Participants reported that the MECA were easy to use, that the MECA helped them feel better prepared, and that they would use the MECA if presented with these medical emergencies in real life. The only significant difference was found in the evaluation of the checklist regarding the reduction of the stress level: “medical” rated a higher score on the 5-scale-likert range than “perioperative” residents (3.7 ± 1.2 vs. 2.9 ± 1.2, *p* < 0.05). Detailed information is given in Table 3.

**Table 3:** Participants’ perceptions of medical emergency cognitive aids (MECA)

	Overall	Perioperative		Medical			*p*-value^§^
Participants		Anesthesia	Surgery	Internal Medicine	Neurology	Others	
N = 501*		85	46	335	28	7	
The most important points are included	4.3 (± 0.81)	4.23 (± 0.99)	4.47 (± 0.63)	4.28 (± 0.79)	4.33 (± 0.75)	4.43 (± 0.53)	**0.38**
I would want the MECA to be used in my intensive care unit	4.2 (± 1.01)	4.1 (± 0.96)	4.03 (± 1.15)	4.17 (± 1.04)	4.44 (± 0.7)	4.57 (± 0.53)	**0.17**
The MECA was precise, understandable and easy to use	4 (± 0.85)	4 (± 0.75)	4.13 (± 0.67)	3.96 (± 0.87)	3.53 (± 1.12)	3.83 (± 0.41)	**0.15**
The MECA provides the required information, so that the emergency scenario can be reliably mastered	4 (± 0.86)	3.9 (± 0.92)	4.03 (± 0.67)	3.91 (± 0.87)	4.17 (± 0.71)	4.43 (± 0.79)	**0.47**
The MECA can be edited in the available time	3.5 (± 0.96)	3.6 (± 1.04)	3.53 (± 0.82)	3.45 (± 0.96)	3.77 (± 0.81)	3.8 (± 1.1)	**0.18**
The MECA helped me feel better prepared during the emergency scenario	3.4 (± 1.01)	3.28 (± 1.11)	3.53 (± 1.1)	3.39 (± 0.97)	3.29 (± 1.1)	4 (± 0.71)	**0.47**
Using the MECA created a relaxed atmosphere among the participants	3.1 (± 1.15)	2.85 (± 1.23)	3.27 (± 1.23)	3.09 (± 1.12)	3.28 (± 1.02)	3.83 (± 1.17)	**0.21**
I felt less stressed in the scenarios when the MECA was available	3.1 (± 1.18)	2.7 (± 1.16)	3.2 (± 1.06)	3.17 (± 1.17)	3.18 (± 1.29)	4.14 (± 1.07)	**0.02**
Problems with the clinical flow or processes that were not considered occurred, so that the usage of the MECA had to be waived	2.8 (± 1.24)	2.89 (± 1.29)	2.61 (± 1.33)	2.72 (± 1.22)	3.06 (± 1.25)	2.76 (± 0.82)	**0.39**
Despite using the MECA, the emergency scenario cannot be reliably mastered	2.6 (± 1.1)	2.48 (± 1.22)	2.87 (± 1.12)	2.6 (± 1.04)	2.88 (± 1.32)	2.66 (± 1.5)	**0.48**

Bold values indicate a* p*-value < 0.05 was considered as statistically significant

^*^Total number of responding participants, number of answers may vary

^§^p was calculated with t-test for “medical” and “perioperative”

## Discussion

In medicine, different data exist on the effectiveness of checklists and MECA: whereas prior research in the perioperative setting showed a highly significant reduction of missed critical process steps (6% when checklists were available vs. 23% when they were unavailable) [3], the implementation of a pediatric sedation safety checklist failed to show a significant reduction in sedation-related adverse events [11]. Additionally, there is conflicting evidence for the effectiveness of checklists to improve perioperative outcomes in some populations [12]. In intensive and emergency care medicine, the implementation of a multidisciplinary safety checklist during bedside bronchoscopy-guided percutaneous tracheostomy was independently associated with a 580% reduction in adverse procedural events [13] and the implementation of a preintubation checklist for ED intubation of trauma patients was associated with a 7.7% absolute risk reduction [14]. Implementation of a multifaceted quality improvement intervention with daily checklists, goal setting, and clinician prompting did not reduce in-hospital mortality among critically ill patients treated in ICUs in Brazil [15]. Since data on risk reduction using checklists in intensive care and emergency medicine still seem to be very limited, it is difficult to classify our results precisely [5]. In order to correctly classify the observed effect, it depends on the intention to treat population. If MECA were not applied, or only partially applied, or in the worst case, the wrong MECA was chosen (expectably leading to worse results than in the control group), this is a very crucial consideration in the evaluation of the system. A per protocol analysis may be helpful to further break down the observed effect. Additionally, checklists may be designed as diagnostic (Scriven schema) or problem-solving (Higgins and Boorman schema), but, as the text-based algorithms used in this study do not contain any obvious actions or criteria to “check off”, we chose the term “medical emergency cognitive aid” (MECA) instead of checklist in order to ensure transparency with readers [16]. In our simulations, MECA were subjectively judged as helpful by 94% of the participants. Overall, our results suggest that in a high-fidelity simulation MECA use by residents of different specialties led to a relevant risk reduction in the management of medical emergencies. Teams performed significantly better when MECA were available even though the MECA used were previously unknown to the study participants. In case of insufficient performance using MECA, those teams have simply refrained from using the checklists to the extent provided. Interestingly the use of wrong MECA led to an even worse performance than not using MECA at all, which resulted from a consecutive fault after misinterpretation of the underlying cardiac rhythm. Our study participants, mostly 2nd or 3rd year residents, were quite equally distributed in terms of age, gender and overall work experience. “Perioperative” residents had significantly more pre-experience in ED medicine and pre-hospital trauma life support (PHTLS), which is in line with previous data from Germany [17]. Participants’ perceptions of MECA revealed a high level of acceptance across all specialties with a single exception regarding potential stress reduction which was significantly more obvious in “medical” versus “perioperative” residents. The underlying cause mainly remains uncertain, and we can only speculate on the reasons. With the introduction of the WHO surgical checklist in 2008 [18], checklists have become an indispensable part of daily surgical and anesthesiologic routine. Thus, maybe “perioperative” residents were more experienced in the overall checklist or MECA use. Additionally, “perioperative” residents had significantly higher preexisting emergency medicine experience, which could also contribute to the fact that the stress-reducing effect in this group was not as pronounced as in the group of "medical" residents.

Participants with prior experience using checklists who were not randomized to the MECA group also rated them mostly favorably.Although our results are promising and inspiring concerning a broader introduction of MECA in hospitals, their use can also be problematic when applied to clinical problems that require nonlinear responses [5] and there may be a risk of therapeutic misalignment [5–8] with the delivery of excess or inappropriate interventions, like in sepsis. To minimize this bias, we deliberately selected established guidelines that can easily be described by checklists, algorithms, MECA or protocols [16–20]. If teamwork-training initiatives are combined with the implementation of MECA, this may confound the results [7]. In our study, MECA—although available—were generally not used in 34% of cases. In the per protocol analysis, MECA were used in 63 of the 120 scenarios. We can only speculate why this was the case despite the broad fundamental agreement in the survey conducted afterwards. With regard to the approximately 50% usage in the intervention group, it is hard to reconcile the 94% of the 501 respondents who subjectively judged the checklists to be useful. We cannot exclude a bias in this context. One has to bear in mind that self-reported perceptions are weaker compared with other more objective forms of evidence related to the impact and efficacy of MECA. However, we anticipate that training the teams with their individual roles within the teamwork before the emergency and, most importantly, training the teams in the handling of the MECA would have improved MECA utilization and may have led to a further reduction in the failure of critical work steps so that our results are potentially underestimated. In fact, the only information our participants received before the start of the scenario was that a total of 10 MECA would be available. In order not to distort the results, the issue of specific MECA content was not broached in advance. In our analysis of the performances of the two groups, we excluded teams who selected to use checklists, but nonetheless, selected the wrong ones during the simulation case scenarios. By doing so, there was a slight chance that this omission of data would inadvertently inflate the ratings of performances of the intervention group. Indeed, a recalculation showed only negligible differences. One might discuss how far the “critical steps” selection process contributes to the construct validity of the ratings based on the scores generated. We aimed to provide a scoring system as transparent and comprehensible as possible and thus chose a binary technique as this method seemed to be most appropriate. Additional limiting factors for MECA usage are lack of time and high stress levels in an emergency. This might explain why most groups used the MECA only brief at the beginning, whereas other groups did not use MECA until they were stuck. Only 10 groups systematically worked through the MECA from the beginning to the end, resulting in > 90% execution of correct work steps.

Simulator-based studies nearly always lack real patients. However, high-fidelity simulation has become an accepted part of medical training and evaluation [3]. Overlooking other fields, like aviation, show that simulation is an established and efficient part of testing and assessing (a) the value of safety protocols and (b) possible consequences in case of deferring from such protocols without the often-deleterious results seen in real life. In case of medical simulation, high agreement rates with findings in real cases have been reported [21, 22]. Simulation also helps to make rare events, like the emergencies used in this study, trainable and enabling to investigate topics that for a variety of practical and ethical reasons would be very difficult to investigate in real cases. Further strengths include the number of participants and the identical conditions for all teams which would have been impossible to achieve outside of a simulated setting. Video-analysis and the high number of checkpoints enabled us to perform a detailed analysis. We are sure about a benefit for trainees when using MECA, however with the numbers as low as they are, that is impossible to say with certainty. Although this may not really be generalizable by now, our study contributes its part to literature, and paves the way for further investigations with additional data and analysis.

## Limitations

One might fear that the use of MECA is potentially contrary to the physician's "therapeutic freedom" and sometimes considered as criticism of one's own competence. Clinicians may feel restricted in their available responses [8]. Strategies to overcome this and further barriers have already been introduced [19]. In the design of our research, we chose to place the MECA in the hands of the frontline providers, where cognitive load is greatest. Another approach might be considering the effect or impact of placing this tool in the hands of another team members (e.g., a nurse in the role of recorder). Many RRT are comprised of interprofessional team members; removing this tool from the "front-line" healthcare provider and placing it in the hands of someone engaged in the response but tasked with a different role. This might be an interesting next direction to consider. Again, this approach might yield an even better demonstration of outcomes associated with MECA use than observed in this study.

It would have been interesting to examine both correctness and efficiency. For this reason, response (processing) time could have been explored between groups to better reflect possible benefits of MECA as well. Due to strict timey regulations we have chosen against temporal measures in the presented study, since no differences were expected.

The error rate of critical work processes, our primary endpoint, may be discussable in the context whether the absolute or relative risk reduction is of primary interest or likewise whether it is the effect in the intention to treat or in the per protocol population. The latter point can actually be answered clearly, since according to all guidelines the ITT population should be the primary evaluation population in a superiority trial. Considering this, still an effect, though less pronounced, was observable. Not being able to blind the video analysist could have affected the review process. However, as only critical care process steps were counted, the influence of a non-blinded analyst should be considered to be lower at most.

Finally, it would have been desirable to be able to test not only the occupational groups but also the two study arms among themselves for equal distribution. However, due to the anonymization requirements, an exact assignment of the respondents to the study arms was unfortunately impossible.

## Conclusions

In our simulation, the use of MECA in medical emergency situations significantly reduced failure rates. The use of MECA was widely accepted, and MECA were easy to use. In a high percentage, stress level of the participants was diminished. Based on our data, an introduction of MECA at least for the ED, the intensive care unit and the in-hospital emergency response teams could be considered. Additionally, MECAs could be used beforehand as teaching aids to avoid potentially fatal misinterpretations in an emergency. Further studies should focus on the transferability to the pre-clinical sector or high-risk in-hospital settings (e.g., catheter laboratory) and their impact on the performance in key aspects (e.g., more benefit and less harm for patients). MECA may lead to a destructive outcome. Therefore, one of the priorities in MECA/checklist implementations in the future should be how to secure the correct cognitive aid selection for a critical incident.

## Supplementary Information

**Additional file 1**. Appendix A.

## Data Availability

The datasets used and/or analysed during the current study are available from the corresponding author on reasonable request.

## Abbreviations

MECA: Medical emergency cognitive aid
RRT: Rapid response team
ED: Emergency department
VT: Ventricular tachycardia
SD: Standard deviation
ICU: Intensive care unit
CPR: Cardiopulmonary resuscitation
MI: Myocardial infarction
PHTLS: Prehospital trauma life support
WHO: World Health Organization

## References

[1] Bernhard M, Döll S, Hartwig T, Ramshorn-Zimmer A, Yahiaoui-Doktor M, Weidhase L, Petros S, Gries A (2018). Resuscitation room management of critically ill nontraumatic patients in a German emergency department (OBSERvE-study). Eur J Emerg Med.

[2] Young AK, Maniaci MJ, Simon LV, Lowman PE, McKenna RT, Thomas CS, Cochuyt JJ, Vadeboncoeur TF (2020). Use of a simulation-based advanced resuscitation training curriculum: impact on cardiopulmonary resuscitation quality and patient outcomes. J Intensive Care Soc.

[3] Arriaga AF, Bader AM, Wong JM, Lipsitz SR, Berry WR, Ziewacz JE, Hepner DL, Boorman DJ, Pozner CN, Smink DS, Gawande AA (2013). Simulation-based trial of surgical-crisis checklists. N Engl J Med.

[4] Wolfe H, Zebuhr C, Topjian AA, Nishisaki A, Niles DE, Meaney PA, Boyle L, Giordano RT, Davis D, Priestley M, Apkon M, Berg RA, Nadkarni VM, Sutton RM (2014). Interdisciplinary ICU cardiac arrest debriefing improves survival outcomes*. Crit Care Med.

[5] Delaney A, Hammond N, Litton E (2020). Checklists and protocols in the ICU: less variability in care or more unnecessary interventions?. Intensive Care Med.

[6] Tang R, Ranmuthugala G, Cunningham F (2014). Surgical safety checklists: a review. ANZ J Surg.

[7] de Jager E, McKenna C, Bartlett L, Gunnarsson R, Ho YH (2016). Postoperative adverse events inconsistently improved by the world health organization surgical safety checklist: a systematic literature review of 25 studies. World J Surg.

[8] Kavanagh BP, Nurok M (2016). Standardized intensive care. Protocol misalignment and impact misattribution. Am J Respir Crit Care Med.

[9] Martinez W, Lehmann LS (2013). The "hidden curriculum" and residents' attitudes about medical error disclosure: comparison of surgical and nonsurgical residents. J Am Coll Surg.

[10] Healy JM, Davis KA, Pei KY (2018). Comparison of internal medicine and general surgery residents' assessments of risk of postsurgical complications in surgically complex patients. JAMA Surg.

[11] Kahlenberg L, Harsey L, Patterson M, Wachsberger D, Gothard D, Holder M, Forbes M, Tirodker U (2017). Implementation of a modified WHO pediatric procedural sedation safety checklist and its impact on risk reduction. Hosp Pediatr.

[12] O’Leary JD, Wijeysundera DN, Crawford MW (2016). Effect of surgical safety checklists on pediatric surgical complications in Ontario. CMAJ.

[13] Hazelton JP, Orfe EC, Colacino AM, Hunter K, Capano-Wehrle LM, Lachant MT, Ross SE, Seamon MJ (2015). The impact of a multidisciplinary safety checklist on adverse procedural events during bedside bronchoscopy-guided percutaneous tracheostomy. J Trauma Acute Care Surg.

[14] Smith KA, High K, Collins SP, Self WH (2015). A preprocedural checklist improves the safety of emergency department intubation of trauma patients. Acad Emerg Med.

[15] Cavalcanti AB, Bozza FA, Machado FR, Salluh JI, Campagnucci VP, Vendramim P, Guimaraes HP, Normilio-Silva K, Damiani LP, Romano E, Carrara F, Diniz L, de Souza J, Silva AR, Ramos GV, Teixeira C, Brandão da Silva N, Chang CC, Angus DC, Berwanger O, Writing Group for the CHECKLIST-ICU Investigators and the Brazilian Research in Intensive Care Network (BRICNet) (2016). Effect of a quality improvement intervention with daily round checklists, goal setting, and clinician prompting on mortality of critically Ill patients: a randomized clinical trial. JAMA.

[16] Chaparro A, Keebler JR, Lazzara EH, Diamond A (2019). Checklists: A review of their origins, benefits, and current uses as a cognitive aid in medicine. Ergonomics in design. Ergon Design Q Human Fact Appl.

[17] Ilper H, Kunz T, Walcher F, Zacharowski K, Byhahn C (2013). An online emergency physician survey–demography, education and experience of German emergency physicians. DMW.

[18] WHO’s patient-safety checklist for surgery (2008). Lancet.

[19] Bergs J, Lambrechts F, Simons P, Vlayen A, Marneffe W, Hellings J, Cleemput I, Vandijck D (2015). Barriers and facilitators related to the implementation of surgical safety checklists: a systematic review of the qualitative evidence. BMJ Qual Saf.

[20] Lott C, Truhlář A, Alfonzo A, Barelli A, González-Salvado V, Hinkelbein J, Nolan JP, Paal P, Perkins GD, Thies KC, Yeung J, Zideman DA, Soar J (2021). ERC special circumstances writing group collaborators. European resuscitation council guidelines 2021: cardiac arrest in special circumstances. Resuscitation.

[21] Weichert V, Sellmann T, Wetzchewald D, Gasch B, Hunziker S, Marsch S (2015). Two minutes CPR versus five cycles CPR prior to reanalysis of the cardiac rhythm: a prospective, randomized simulator-based trial. Resuscitation.

[22] Vogt L, Sellmann T, Wetzchewald D, Schwager H, Russo S, Marsch S (2020). Effects of bag mask ventilation and advanced airway management on adherence to ventilation recommendations and chest compression fraction: a prospective randomized simulator-based trial. J Clin Med.

