# Supplementary material for: Simulation-based randomized trial of medical emergency cognitive aids
Source: Scand J Trauma Resusc Emerg Med. 2022 Jul 11;30:45. doi: 10.1186/s13049-022-01028-y (PMC9277856; doi:10.1186/s13049-022-01028-y)
Supplement: Supplementary file 1 — Additional file 1. Appendix A. [file 13049_2022_1028_MOESM1_ESM.docx]

**Appendix A** Medical emergency cognitive aids (MECA) available for scenarios

**9 - Ventricular tachycardia – hemodynamically unstable**

*Persistent tachycardia with hypotension, chest pain, confusion or shock*

**Start**

1. **Call for help, use checklist**
   - Who is team leader?^1^

**Critical change**

## Set FiO_2_ to 100%^2^

If cardiac arrest, then go to

- Checklist 10 Asystole/PEA
- Checklist 5 VF/VT

1. **Rhythm analysis**
   - Irregular and broad complex tachycardia

-> treat like VF (> Checklist 5)

- - Regular broad complex tachycardia and narrow complex tachycardia -> cardioversion

## Cardioversion

- - Get defibrillator (Zoll M or R series) (emergency trolley)
  - Sedate and analgize awake patients^3^

**During cardioversion**

- - Switch on defibrillator (Zoll M or R series)
  - Place AED electrodes or apply AED patches with gel and press firmly

Safe airway? Safe venous access? ^7^

- - **Activate Sync**^4^
  - Select energy (pushbuttons)

- 1^st^ attempt 100 J^5^, 2^nd^ attempt 150 J^6^, 3^rd^ attempt 200 J

- - Press "Charge" button and, after a security check, enable power; Keep button pressed until energy is released^8^

VF = ventricular fibrillation; VT = ventricular tachycardia; AED = automatic external defibrillator; PEA = pulseless electric activity;

Red marked work steps indicate the ones that were evaluated, increase of cardioversion energy was counted as one step each

#
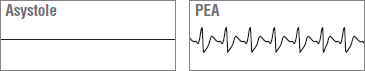
10 Cardiac arrest - asystole/ PEA (pulseless electrical activity)

*Pulseless cardiac arrest that cannot be terminated by an electric shock*

**Start**

| **Medication: Dosage** |
| --- |
| Epinephrine: 1 mg every 3-5 min  ----------------------------------------------------------------  **Intoxication with**  Beta blockers Glucagon 2-4 mg  Ca-Antagonist Calcium 1 g  Local anesthetics MCT solution 20 %  (Bolus 1,5 ml/kg, then 0,1 ml/kg/min for 30 min)  ----------------------------------------------------------------  **Hyperkalemia**   - Ca-Gluconate 30 mg/kg *or* - Ca-Chloride 10 mg/kg - If pH<7,2 NaHCO3 50 ml - Insulin 10 IU + Glucose 20 % |
| **4Hs’ & 4Ts’** |
| -**H**ypoxia^8^ -**T**ension pneumothorax^8^  -**H**ypovolemia^8^ -**T**amponade^8^  -**H**ypothermia^8^ -**T**oxic substances^8^  -**H**yperkalemia -**T**hromboembolism |
|  |

1. **Call for help, use checklist**
   - Who is team leader?^1^
   - Command: "CPR has absolute priority"
2. **Place patient on back on a hard surface**^2^
3. **Set FiO_2_ to 100%**
4. **Start CPR according to ERC guidelines**
   - “Hard and fast” chest compressions, 100/min.^3^
   - Allow complete chest decompression^4^
   - Chest compression: manual ventilation = 30:2 (if airway unsecured)^5^

## Epinephrine application 1 mg as soon as possible^6^; then every 3-5 min

1. **Evaluation every 2 min.**
   - Change of helpers for chest compression^7^
   - Rhythm analysis
     - If asystole/PEA persists:
       - Go back to 4)

| **During CPR** |
| --- |
| Safe airway? Safe venous access? Distribution of tasks: chest compression, airway, venous access, checklist, time control, documentation |

- - - - Read out the 4Hs’ and 4Ts’ loudly (Box on the right)
    - If VF/VT, go to > checklist 7

CPR = cardiopulmonary resuscitation; ERC = European Resuscitation Council; VF = ventricular fibrillation; VT = ventricular tachycardia; MCT = medium chain triglycerides; NaHCO3 = Sodium bicarbonate. Red marked work steps indicate the ones that were evaluated; 4 Hs`& 4Ts`counting as one work step (1 critical point)


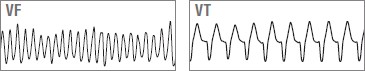
**7 - Cardiac Arrest - VF/ pVT**

*Defibrillable pulseless cardiac arrest*

**Start**

**Medication: Dosage**

Epinephrine: 1 mg every 3-5 min

----------------------------------------------------------------

**Antiarrhythmics**

Amiodarone 300 mg, 2nd dose 150 mg Magnesium 1-2 g if Torsade de Pointes

### Call for help, use checklist

- - Who is team leader?^1^
  - Announcement: "*Defibrillation as soon as the defibrillator is connected."*

### Place patient on back on a hard surface^2^

### Set FiO_2_ to 100%

**Defibrillation**

1. **Start CPR according to ERC guidelines**
   - Hard and fast chest compressions 100/min^3^

- Defibrillator located on trolley (Zoll M or R series)
- Switch on Defibrillator
- Use fast patch electrodes if available
- Select energy (preset 120J, increase to 200J)
  - Allow chest to decompress completely^4^
  - Chest compression: manual ventilation = 30: 2 (if airway unsecured)^5^

### Defibrillation

| **4Hs’ & 4Ts’** | | |
| --- | --- | --- |
| -**H**ypoxia^8^ | - **T**amponade^8^ |  |
| -**H**ypovolemia^8^  -**H**ypothermia^8^ | - **T**ension pneumothorax^8^  - **T**hromboembolism^8^ |  |
| -**H**ypo-/ Hyperkalemia^8^ | Coronary/ pulmonary^8^  - **T**oxic substances^8^ |  |
|  | r |  |
|  |  |  |

- - 200 J, fast patches preferred, paddles with gel and press firmly
  - Restart CPR immediately after shock delivery; next rhythm analysis

after 2 min

### Epinephrine application 1 mg every 3-5 min

1. **Consider antiarrhythmics (amiodarone) after 3rd defibrillation**^6^
2. **Evaluation every 2 min.**
   - Change of personnel for chest compression^7^

**During CPR**

- - Rhythm analysis
  - If VT/VF persists, go back to 4)

Safe airway? Safe venous access? Distribution of tasks: chest compression, airway, venous access, checklist, time control, documentation

- - Read out the 4 Hs’ and 4Ts’ loudly (box on the right)
  - If Asystole/PEA, go to > checklist 10

CPR = cardiopulmonary resuscitation; ERC = European Resuscitation Council; VF = ventricular fibrillation; pVT = pulseless ventricular tachycardia; PEA = pulseless electric activity. Participants received a folder containing a total of 10 checklists in German language for use during three simulated life-threatening medical emergencies. Red marked work steps indicate the ones that were evaluated; 4 Hs`& 4Ts`counting as one work step (= 1 critical point).
